# Integrating multi-modal data fusion approaches for analysis of dairy cattle vocalizations

**DOI:** 10.3389/fvets.2025.1704031

**Published:** 2025-11-17

**Authors:** Bubacarr Jobarteh, Madalina Mincu-Iorga, Dinu Gavojdian, Suresh Neethirajan

**Affiliations:** 1 Faculty of Computer Science, Dalhousie University, Halifax, NS, Canada; 2 Cattle Production Systems Laboratory, Research and Development Institute for Bovine, Balotesti, Romania; 3 Faculty of Agriculture, Agricultural Campus, Dalhousie University, Truro, NS, Canada

**Keywords:** acoustic pattern analysis, bioacoustics monitoring, cattle vocalizations, multi-modal data fusion, precision livestock farming

## Abstract

Non-invasive analysis of dairy cattle vocalizations offers a practical route to continuous assessment of stress and timely health interventions in precision livestock systems. We present a multi-modal AI framework that fuses standard acoustic features (e.g., frequency, duration, amplitude) with non-linguistic, transformer-based representations of call structure for behavior classification. The classification analysis represents the core contribution of this work, while the integration of the Whisper model serves as a complementary exploratory tool, highlighting its potential for future motif-based behavioral studies. Using contact calls recorded from a cohort of lactating Romanian Holsteins during a standardized, brief social-isolation paradigm, we developed an ontology distinguishing high-frequency calls (HFCs) associated with arousal from low-frequency calls (LFCs) associated with calmer states. Across cross-validated models, support vector machine and random-forest classifiers reliably separated call types, and fused acoustic + symbolic features consistently outperformed single-modality inputs. Feature-importance analyses highlighted frequency, loudness, and duration as dominant, interpretable predictors, aligning vocal patterns with established markers of arousal. From a clinical perspective, the system is designed to operate passively on barn audio to flag rising stress signatures in real time, enabling targeted checks, husbandry adjustments, and prioritization for veterinary examination. Integrated with existing sensor networks (e.g., milking robots, environmental monitors), these alerts can function as an early-warning layer that complements conventional surveillance for conditions where vocal changes may accompany pain, respiratory compromise, or maladaptive stress. While the present work validates behaviorally anchored discrimination, ongoing efforts will pair vocal alerts with physiological measures (e.g., cortisol, infrared thermography) and multi-site datasets to strengthen disease-specific inference and generalizability. This framework supports scalable, on-farm welfare surveillance and earlier intervention in emerging health and stress events.

## 1 Introduction

Vocal signals play a central role in social and emotional expression across the animal kingdom. Mounting empirical evidence demonstrates that a cow’s emotional and physiological state is reliably mirrored in its vocal behavior (1). Specifically, acoustic structures such as frequency, amplitude, duration, and vocalization rate vary systematically in response to emotional arousal. For example, heightened arousal and distress states often lead to vocalizations that are louder, longer, and higher in pitch. Conversely, contentment or affiliative interactions are typically accompanied by softer, shorter, and lower-frequency calls (2, 3). This predictable variation makes vocalization analysis a powerful tool for automated, objective welfare assessments that can complement subjective observational methods. In dairy cows, vocal signals can be broadly categorized into high-frequency calls (HFCs) and low-frequency calls (LFCs), each associated with distinct behavioral and emotional contexts. HFCs are generally linked to situations of arousal, agitation, isolation, or discomfort. These calls are often emitted at higher intensities and serve long-distance communicative functions, especially under distress (4, 5). LFCs, on the other hand, are commonly produced during relaxed, affiliative, or social bonding contexts. These low-frequency sounds are typically made at close proximity and are often indicative of positive emotional valence, being produced particularly in cow-calf interactions (6, 7). However, such associations remain context-dependent and should not be interpreted as direct indicators of valence. Housing systems, climatic conditions, ambient noise, and herd density can all influence the type, frequency, and amplitude of vocalizations. For instance, cows housed on pasture have been observed to vocalize differently compared to those in confined indoor settings, likely due to increased opportunities for natural behaviors and social engagement (8). Acoustic properties of the environment, such as reverberation and background noise levels, also were shown to modulate vocal behavior. The present study was built on these foundations by integrating multi-source data fusion and advanced computational models to decode dairy cow vocalizations in a negative emotional state context. At the core of the methodological innovation is the use of the Whisper model, a transformer-based acoustic representation tool developed by OpenAI (9). Although originally designed for human speech recognition, Whisper has demonstrated remarkable adaptability to noisy, unstructured bioacoustics data (10, 11). Praat was used for acoustic feature extraction, while Whisper was applied to detect symbolic motifs, providing complementary insights and practical robustness in barn-noise conditions. This approach is analogous to the use of spectrograms as visual tools that facilitate frequency-time domain analysis. By using Whisper-derived sequences, this work was able to generate a text-like symbolic form that simplifies the extraction of recurring motifs, such as bigrams or trigrams, that may correlate with specific emotional states. Worth mentioning is that “bigram” and “trigram” counts are used here purely as statistical descriptors of token adjacency, commonly applied for motif discovery in animal vocal sequences, and do not imply grammatical structure. Similar approaches have been employed in studies on primates and birds to identify combinations of acoustic elements associated with affective or contextual meaning (12). Among the various features extracted from cow vocalizations, frequency and amplitude consistently emerge as the most informative (13, 14). Frequency is particularly sensitive to changes in emotional arousal, often increasing during heightened stress or isolation events. Amplitude reflects the intensity or urgency of a call, with louder sounds typically associated with more acute states of discomfort. Duration and vocalization rate further enrich this analysis by providing temporal dynamics that can differentiate between chronic and transient stressors. The integration of these features into a unified analytical model, particularly when contextual metadata is available, would allow for more accurate and interpretable classification of emotional states. The theoretical framework underpinning our approach draws from systems biology and evolutionary ethology. Concepts such as degeneracy and modularity are central to understanding how vocal signals can robustly convey affective states despite environmental variability. Degeneracy refers to the phenomenon where multiple different acoustic features can serve overlapping communicative functions. For example, both increased frequency and extended duration may signal distress, providing redundancy that enhances signal reliability (15). Modularity, on the other hand, captures the idea that vocal features can be grouped into functional clusters, such as temporal versus spectral characteristics, that can independently evolve or adapt to contextual demands. From an evolutionary standpoint, the structure of vocalizations in mammals often conforms to Morton’s motivation-structural rules. These rules predict that aggressive or high-arousal calls are typically high-pitched and tonally complex, while affiliative or low-arousal calls tend to be lower-pitched and more harmonically stable (16). This ethological principle has been validated in a range of species, including pigs, goats, and birds (13, 17). Observations in dairy cows suggest that these rules apply similarly, reinforcing the biological plausibility of acoustic classifications (18). To analyze vocal patterns, we employed a suite of machine learning algorithms, including Random Forest, Support Vector Machine (SVM), and Recurrent Neural Networks (RNN). Each of these models bringing distinct strengths to the task of acoustic classification. Random Forest is particularly adept at handling high-dimensional data with mixed feature types, while SVM excels in separating nonlinear classes in sparse datasets. RNNs are uniquely suited for modeling temporal sequences, making them ideal for decoding the structure and dynamics of vocal patterns over time. This study presents a novel multi-modal framework for analyzing cattle vocalizations, integrating acoustic and symbolic features within machine learning classifiers to advance automated behavior classification and real-time welfare monitoring in precision livestock systems. Our hypothesis was that fusing standard acoustic features with Whisper-derived symbolic motifs improves discrimination between high-frequency (HF) and low-frequency (LF) calls compared with single-modality models (acoustic-only or symbolic-only). We formulated the following predictions: (i) model hierarchy: SVM ≥ Random Forest > RNN under our data constraints; (ii) top features: frequency, loudness, and duration will rank highest; (iii) motifs: frequent bigrams (e.g., “rr”) will align with HFC episodes; and (iv) performance: fused features will outperform either modality alone.

## 2 Materials and methods

### 2.1 Study design and data collection

Data were collected at the experimental farm of the Research and Development Institute for Bovine in Balotesti, Romania. The herd was managed indoors year-round under a zero-grazing system, with cows housed in tie-stall barns and provided daily access to outdoor paddocks. We selected 20 multiparous lactating Romanian Holstein cows with homogeneous characteristics in terms of body weight (average 619.5 ± 17.4 kg), lactation stage (II–III), age, and acclimation to housing (minimum 40 days in milk). Our selection criteria targeted physiological and behavioral homogeneity to minimize confounding factors. By including only multiparous cows in mid-lactation with similar body weights, we controlled for anatomical and hormonal variability affecting vocal production traits. Each cow underwent a standardized isolation protocol in which it was visually separated from its herd-mates for 240 min post-milking, being tethered in a 1.8 by 1.2 m stall. Cows were milked twice daily, and all isolation sessions were conducted after the morning milking (7:00–11:00 a.m.) to control for post-milking oxytocin release, circadian rhythm effects, and to ensure consistent daylight recording conditions. During this time, the rest of the herd was relocated to adjacent outdoor paddocks, allowing only auditory contact between individuals. Although occasional background sounds (e.g., distant cow calls, machinery noise, or human activity) were present, the isolated cow’s vocalizations were easily distinguishable and all ambiguous or overlapping signals were manually removed during quality control. The isolation procedure is a widely recognized behavioral paradigm to induce a mild negative affective state (19, 20). To minimize external influences, human access was restricted and machinery activity near the barns was limited. Audio recordings were conducted continuously for the entire 4-h period using high-fidelity equipment: Sennheiser MKH416-P48U3 directional microphones (Sennheiser Electronic GmbH & Co. KG, Wedemark, Germany) mounted on tripods at a distance of 5–6 meters from the cows, connected to Marantz PMD661 MKIII solid-state recorders (Marantz Professional, London, UK). The recordings were captured in WAV format at 44.1 kHz sampling rate and 16-bit resolution. A total of 1,144 vocalizations were retained after rigorous quality control to exclude environmental noise and overlapping signals. The dataset included 952 high-frequency calls (HFCs) and 192 low-frequency calls (LFCs).

### 2.2 Vocalization segmentation and feature extraction

Audio files were segmented into discrete vocalization events using Praat software [v6.0.31; (21)]. Each vocalization was annotated with 23 acoustic features commonly employed in bioacoustic analysis. These included fundamental frequency (F0), duration, amplitude modulation (AMVar, AMRate, AMExtent), formant frequencies (F1–F8), harmonicity, and Wiener entropy (2, 22). The features were selected for their demonstrated relevance to emotional expression in cattle and other mammals. Vocalizations were categorized as either high-frequency calls (HFCs) or low-frequency calls (LFCs) based on spectral thresholds established in prior literature. Vocalizations with dominant peak frequencies above 400 Hz were classified as HFCs, and those below were labeled as LFCs (7). These thresholds are consistent with known differences in vocal tract configuration during high- versus low-arousal states. Table 1 summarizes the acoustic features analyzed in this study, along with their operational definitions and supporting references. The vocalization recordings were analyzed using the Praat DSP package [v.6.0.31; (21)], along with custom-built scripts previously developed by Briefer et al. (23, 24), Reby and McComb (25), Beckers (26), and Briefer et al. (27), to automatically extract the acoustic features for each vocalization.

**Table 1:** Parameters extracted from the cows vocalizations.

Parameter	Definition	References
Duration	The duration of a vocalization.	(17, 27–29)
Vocalization rate	The number of vocalizations in a certain time frame.	(27, 30, 31)
F0	The fundamental frequency and its contour (e.g., min, mean, max and range).	(23, 28, 32, 33)
FMextent	The variation between two peaks of each F0 modulation in Hz.	(23)
Bandwidth	The difference between the highest and lowest observed frequency (Hz).	(32)
Amplitude	Level of energy in the vocalization, the intensity of a vocalization (decibel).	-
AMextent	The mean-to-mean peak variation of each amplitude modulation (decibel).	(28, 34)
AMrate	The number of amplitude modulations in a certain time frame.	(29)
AMVar	The cumulative variation in amplitude divided by the duration of a vocalization (dB/s).	(28)
Q25%	The frequency below which 25 percent of the energy is contained (Hz).	(28, 29, 32, 34)
Q50%	The frequency below which 50 percent of the energy is contained (Hz).	(28, 29, 32)
Q75%	The frequency below which 75 percent of the energy is contained (Hz).	(28, 29, 34)
Formants	Frequencies that correspond to the resonances of the vocal tract.	-
F1mean, F2mean, F3mean, F4mean	The mean frequency of each formant (Hz).	(29)
F1, F2, F3 and F4 range	The frequency range of each formant, thus the difference between the maximum and minimum frequency of that formant (Hz).	(29)
Fpeak	The frequency of peak amplitude.	-

### 2.3 Data preprocessing and representation

All acoustic features were normalized to *z*-scores to account for inter-individual variation. Symbolic sequence representations were generated for each vocalization using the OpenAI Whisper model. This step did not involve linguistic interpretation but served as a means of symbolic encoding to facilitate sequence analysis, such as bigram frequency assessment. This approach has been employed in computational neuroethology to identify recurring motifs in non-human animal communication (12, 35). All audio files were first segmented into discrete vocalization events via Praat, with precise manual annotations. Each segmented vocalization was processed through the Whisper model, which generated symbolic acoustic sequences (e.g., bigram/trigram patterns). These Whisper-derived motifs were time-aligned with acoustic features (e.g., frequency, duration, amplitude, formant measures), enabling correlation and cross-validation. Dominant motifs (such as “rr,” “mm,” “oo”) were empirically mapped to specific spectro-temporal acoustic profiles for each call type—demonstrating direct correspondence between symbolic and conventional bioacoustic parameters. The approach, code, and mapping files are supplied as Supplementary material and openly accessible repository resources. The Python libraries Librosa, NumPy, and Pandas were used for preprocessing and feature extraction. Librosa was particularly instrumental in spectral and temporal feature computation, including pitch tracking and harmonic-to-noise ratio calculations.

### 2.4 Classification models and training

To classify vocalizations, we implemented three machine learning algorithms: Random Forest, Support Vector Machine (SVM), and Recurrent Neural Network (RNN). Random Forest was used for its interpretability and robustness to noise, while SVM provided strong performance in high-dimensional spaces. The RNN model, leveraging temporal dependencies, was suited for detecting patterns in sequential vocal frames. The dataset was split into an 80% training set and 20% testing set, using five-fold cross-validation to assess model performance. Evaluation metrics included accuracy, precision, recall, and F1-score. Feature importance was assessed using permutation techniques in the explainable models, revealing that amplitude-related features (e.g., AMVar, AMRate), spectral entropy, and formant dispersal were among the most informative for classification. The model training workflow is summarized as follows: the RNN architecture consisted of a Long Short-Term Memory (LSTM) network with 128 hidden units, a dropout rate of 0.2, and an Adam optimizer (learning rate = 0.001), using tanh and sigmoid activations for hidden and output layers, respectively. A total of 23 acoustic features extracted via Praat were standardized using z-scores and filtered through correlation analysis, followed by Random Forest importance ranking to retain the top 15 features based on permutation importance, ensuring dimensionality reduction while preserving the most informative predictors for model performance. The computational environment included Python 3.8, TensorFlow 2.8, scikit-learn 1.0.2, Librosa 0.9.1, and Praat 6.0.31. The validation strategy used an 80/20 train–test split with 5-fold cross-validation, and performance was evaluated using accuracy, precision, recall, and F1-score.

### 2.5 Sentiment pattern analysis (exploratory)

As an exploratory extension, bigram frequency analysis on the Whisper-generated symbolic sequences were performed. The goal was to detect recurring acoustic motifs potentially indicative of persistent emotional states. This analysis was inspired by previous motif-based studies in vocal learning species such as songbirds and marmosets (12, 36). Motif selection followed systematic quantitative criteria, including a frequency threshold (>5% of corpus, >57 occurrences), cross-individual consistency (≥15 of 20 cows), and temporal clustering (>60% co-occurrence within specific emotional contexts). Associations with HFC and LFC categories were tested using chi-square (*p* < 0.05). Dominant motifs included “rr” (40,000 + occurrences, linked to rapid modulations in HFCs), “mm” (35,000+, correlated with stable LFC patterns), and “oo” (28,000+, associated with intermediate frequencies). Each motif underwent acoustic–symbolic correlation, spectro-temporal alignment, and cross-validation using independent test subsets.

### 2.6 Cow vocalization ontology and feature mapping

A structured ontology was developed categorizing vocalizations into profiles based on acoustic parameters. High-frequency calls were defined by F0 values between 110.59–494.16 Hz, amplitude between −39.71 to −2.45 dB, and durations from 0.638 to 9.581 s. In contrast, LFCs were characterized by F0 values between 72.61–183.27 Hz, amplitude from −53.88 to −8.16 dB, and durations from 0.650 to 2.921 s. These ranges were consistent with values reported in prior literature (4).

## 3 Results

### 3.1 Acoustic feature analysis

**Figure 1:**
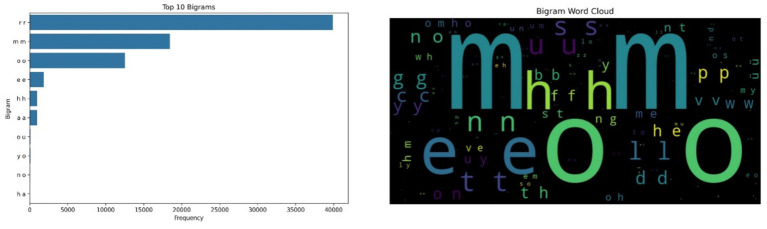
Bigram frequency analysis of symbolic cow vocalizations (left side); Word Cloud of bigram frequencies, where larger text size reflects higher occurrence, with ‘rr’ and ‘mm’ as dominant patterns (right side).

The bigram “rr” emerged as the most frequently occurring symbolic unit, appearing approximately 40,000 times, followed by “mm” and “oo.” These patterns are not interpreted linguistically but rather viewed as symbolic encodings of recurring acoustic shapes produced during vocalizations. The regularity of these bigrams, especially during prolonged vocal episodes, suggests that cows may exhibit rhythmic, repeated vocal behaviors in certain emotional contexts, particularly under emotional distress. Figure 1 illustrates the bigram frequency analysis and the corresponding word cloud.

**Figure 2:**
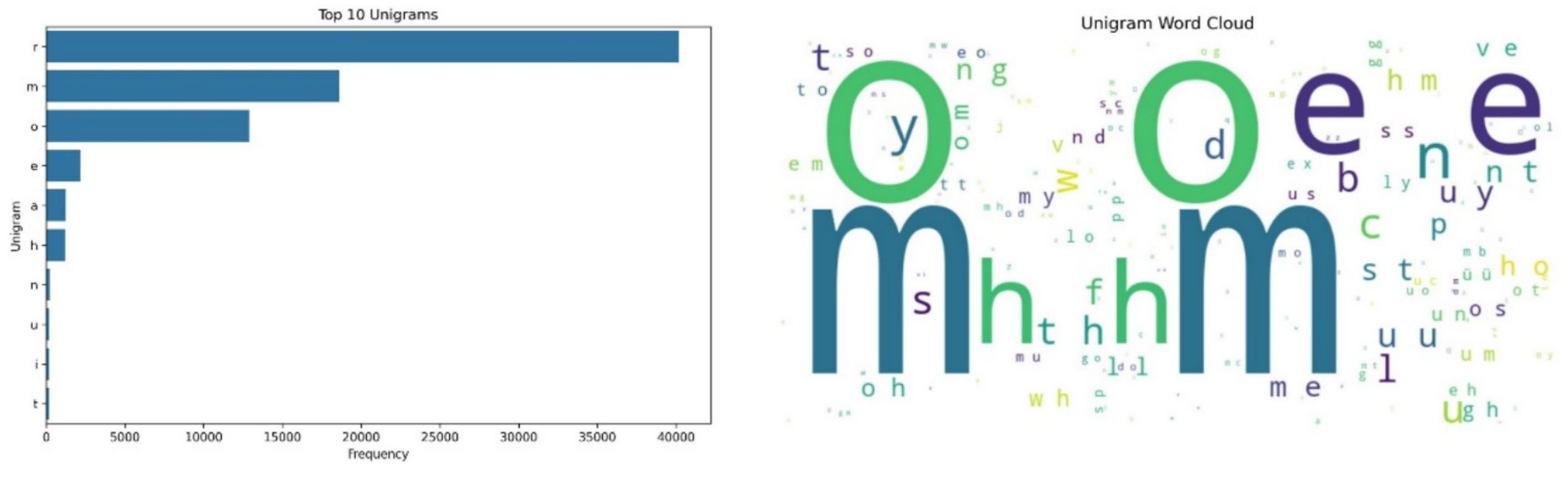
Left side – Unigram Frequency Distribution, showing ‘r’, ‘m’, and ‘o’ as the most frequent characters, indicating consistent symbolic encoding of common vocal elements; Right side – Word Cloud of unigram frequencies, highlighting dominant vocal elements in symbolic form.

Figure 2 displays the results of the unigram analysis and its corresponding word cloud. The character “r” dominates the dataset, followed by “m,” “o,” “e” and “a.” These high-frequency characters appear to reflect consistent symbolic encodings of dominant acoustic patterns, while the less common characters such as “h,” “n” “u,” “i” and “t” point to rarer vocal signatures. These symbolic encodings support the detection of structural diversity in cow vocal expressions and may serve as proxies for repetitive call elements or phonatory modulations. The recurrence of certain unigrams and bigrams, especially in distress-linked HFCs, suggests acoustic motifs that can be incorporated into machine learning pipelines for emotion classification. The acoustic analysis focused on five dimensions—spectral, temporal, amplitude and energy, formant, and prosodic features—using multi-modal fusion. These features were examined in relation to the categorizations: high-frequency calls (HFCs), and low-frequency calls (LFCs).

**Figure 3:**
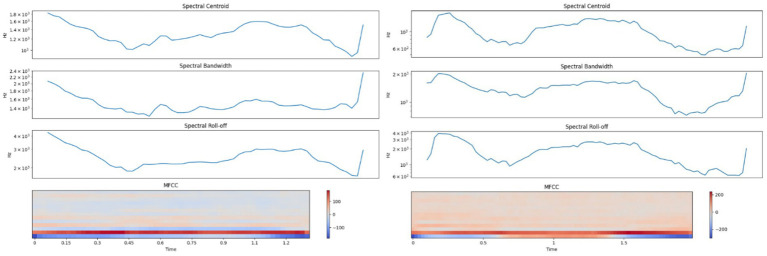
Left side – Spectral analysis of a typical Low-Frequency Call (LFC); Right side – Spectral analysis of a typical High-Frequency Call (HFC).

LFCs exhibited spectral centroids between 1,000–1,800 Hz with energy concentrated below 3,000 Hz, and narrower bandwidths. In contrast, HFCs showed elevated centroids extending from 600 Hz up to 3,000 Hz and spectral energy reaching beyond 4,000 Hz. Mel-frequency cepstral coefficients (MFCCs) were also markedly different: LFCs demonstrated gradual transitions, while HFCs exhibited sharp spectral shifts. These patterns align with prior research on arousal-induced vocal variability (37–39). Figure 3 displays the spectral profiles of LFCs and HFCs.

**Figure 4:**
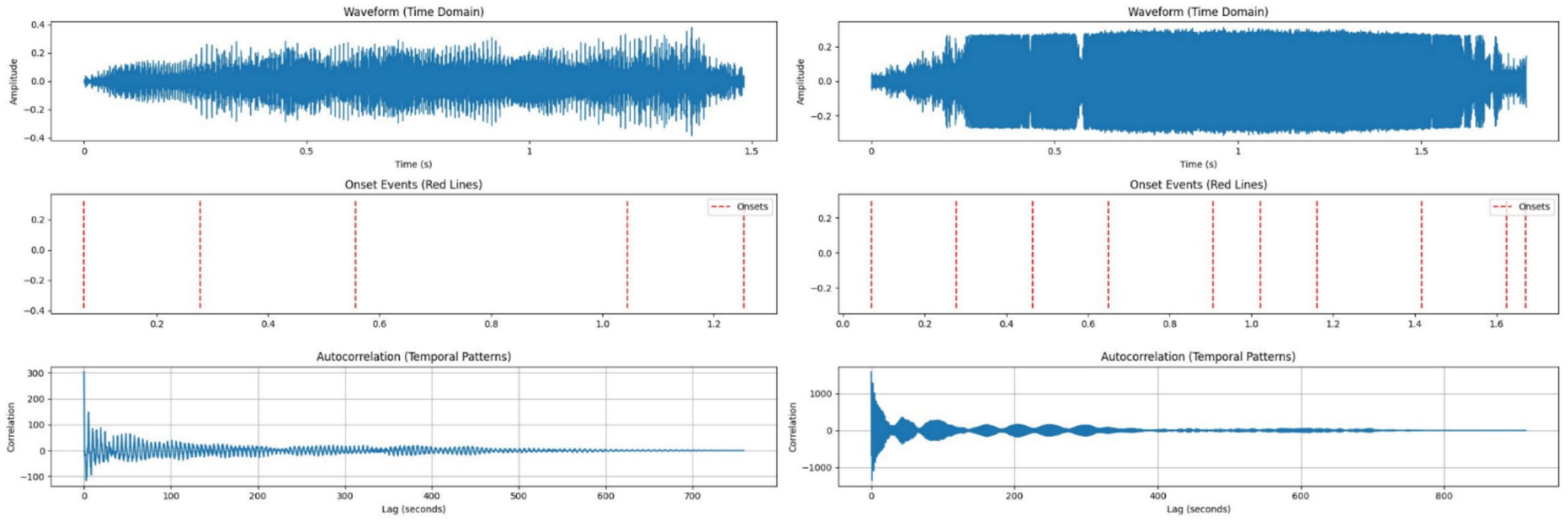
Left side – Temporal analysis of a typical Low-Frequency Call (LFC); Right side – Temporal analysis of a typical High-Frequency Call (HFC).

HFCs averaged 1.79 s with 10 rapid modulations per call and a mean interval of 0.18 s, some as brief as 0.0464 s. LFCs lasted 1.48 s with 5 modulations per call and longer average intervals of 0.30 s, suggesting stable social communication. These observations are consistent with findings by Hernández-Castellano et al. (40) and Gavojdian et al. (22), who reported increased temporal fragmentation in stress-related vocalizations. Figure 4 reveals distinct temporal dynamics between call types.

**Figure 5:**
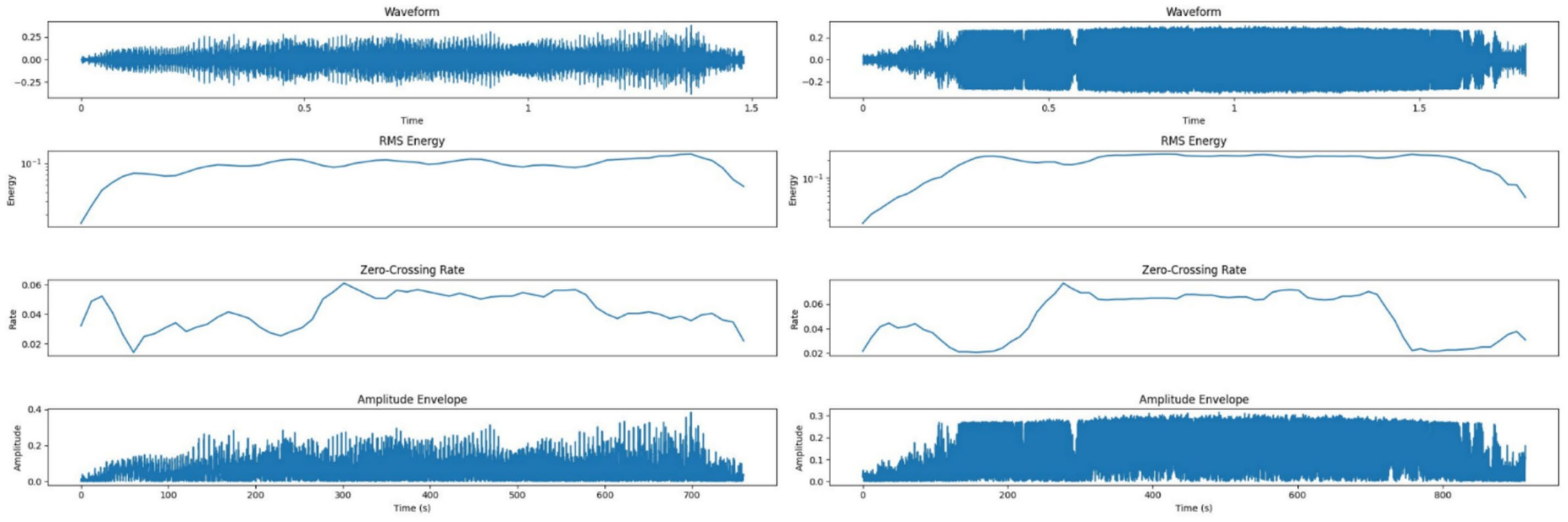
Left side – Amplitude and energy profile of a Low-Frequency Call (LFC), showing lower RMS energy and smoother transitions supporting a calm vocal classification; Right side – Amplitude and energy profile of a High-Frequency Call (HFC), with higher RMS energy and variability reflecting vocal strain and emotional intensity.

Figure 5 shows amplitude and RMS energy patterns for both call types. LFCs had a lower RMS energy mean (0.0934) and lower zero-crossing rate (0.0427), corresponding to smoother transitions. HFCs demonstrated higher RMS energy (0.1887) and a higher zero-crossing rate (0.0492), which can be indicative of rapid vocal shifts and increased acoustic turbulence during emotional arousal (2, 41).

**Figure 6:**
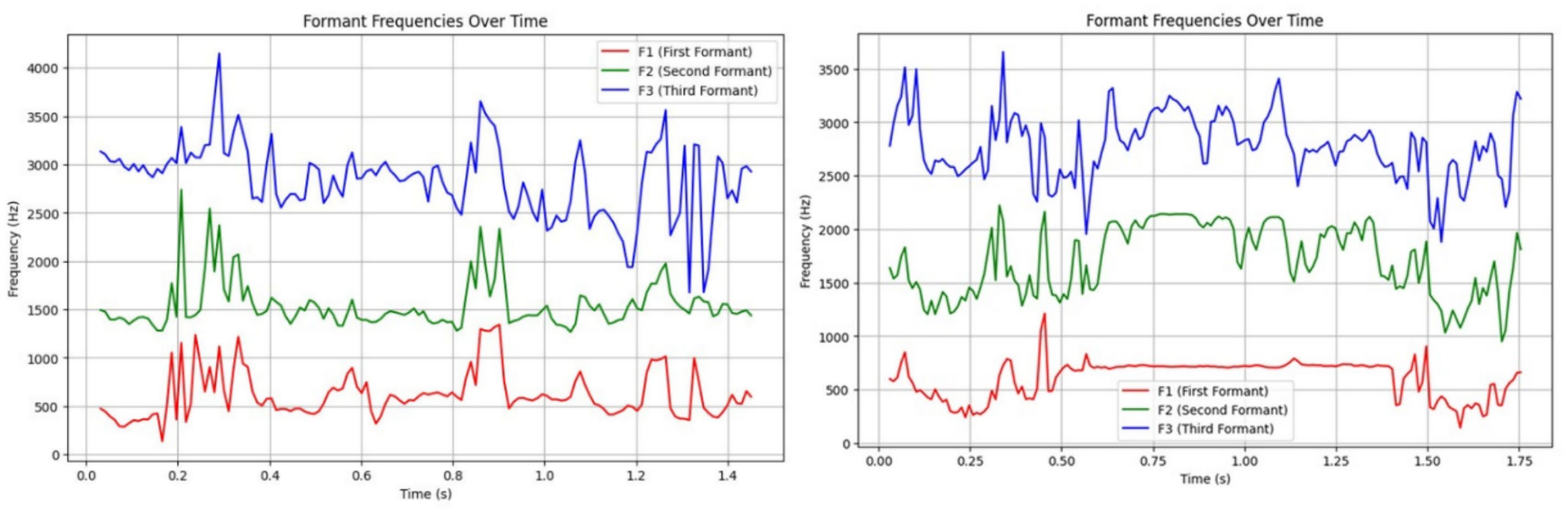
Left side – Formant analysis of a Low-Frequency Call (LFC), where consistent formant structures suggest relaxed vocal tract configurations; Right side – Formant analysis of a High-Frequency Call (HFC), with higher F2 and F3 values indicating vocal tract tension consistent with distress.

Figure 6 compares formant structures of both call types, showing similar F1 values (~617 Hz), but HFCs displayed elevated F2 (1,704.81 Hz vs. 1,542.96 Hz for LFCs), likely reflecting constriction of the vocal tract under emotional stress. LFCs had slightly higher F3 values (2,844.92 Hz vs. 2,779.11 Hz), consistent with a more relaxed vocal tract configuration. These trends support previous findings in vocal source-filter theory applied to affective states (16, 42, 43).

**Figure 7:**
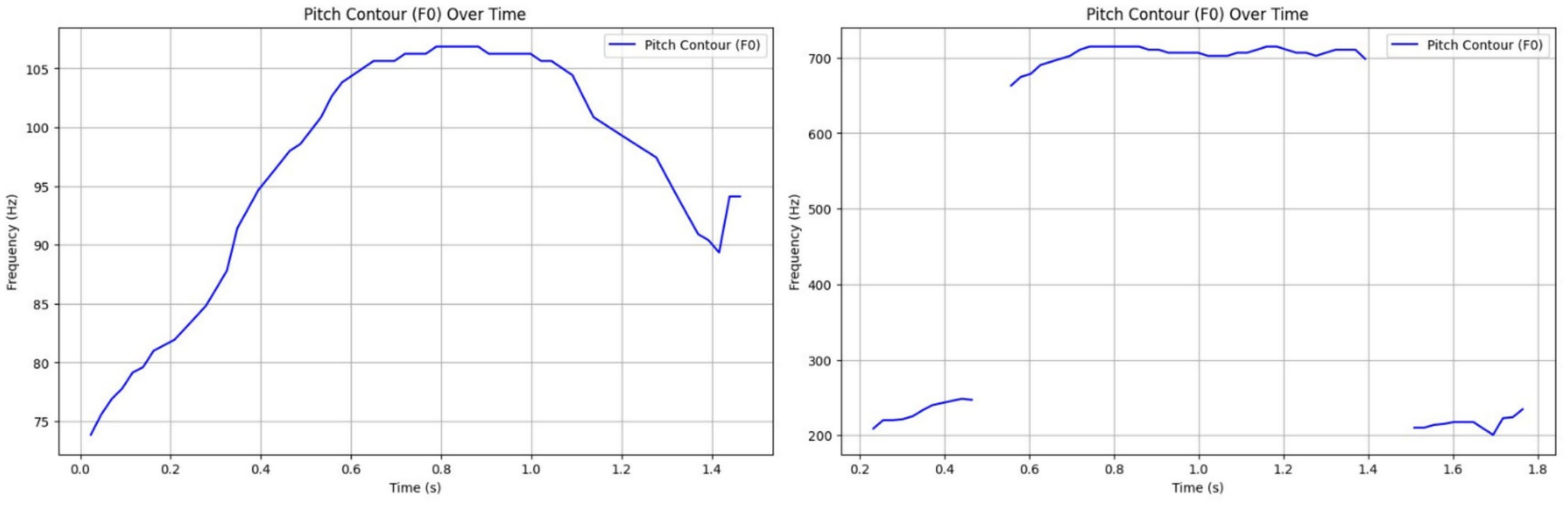
Left side – Prosodic pattern of a Low-Frequency Call (LFC), with a narrow F0 range and stable rhythm indicating calm, affiliative signaling; Right side – Prosodic pattern of a High-Frequency Call (HFC), with a broad F0 range and erratic modulation reflecting stress or agitation.

HFCs presented a broad F0 range of 514.20 Hz, often with erratic pitch and tempo, characteristic of stress or alarm. LFCs had a much narrower pitch range (33.03 Hz), showing tonal consistency and social bonding cues, possible facilitated by the communication with the heard mates from the nearby paddocks (Figure 7). These results are aligned with previous work indicating that prosodic modulation is a key marker of emotional intensity (17, 44).

The acoustic contour analysis revealed clear motif-specific correlations across five dimensions. “rr” motifs were linked to rapid spectral transitions (600–3,000 Hz) and high RMS energy (0.1887 ± 0.05) with frequent amplitude modulations (zero-crossing = 0.0492), whereas “mm” patterns showed stable spectral centroids (1000–1800 Hz), lower RMS energy (0.0934 ± 0.03), and smoother amplitude transitions (zero-crossing = 0.0427). “oo” sequences occupied intermediate frequency ranges with moderate spectral variability. Temporally, “rr” motifs exhibited wider F0 ranges (514.20 Hz) and longer durations (1.79 s), while “mm” motifs appeared in shorter calls (1.48 s) with stable pitch trajectories, confirming distinct acoustic contours aligned with emotional context.

### 3.2 Classification model performance

**Figure 8:**
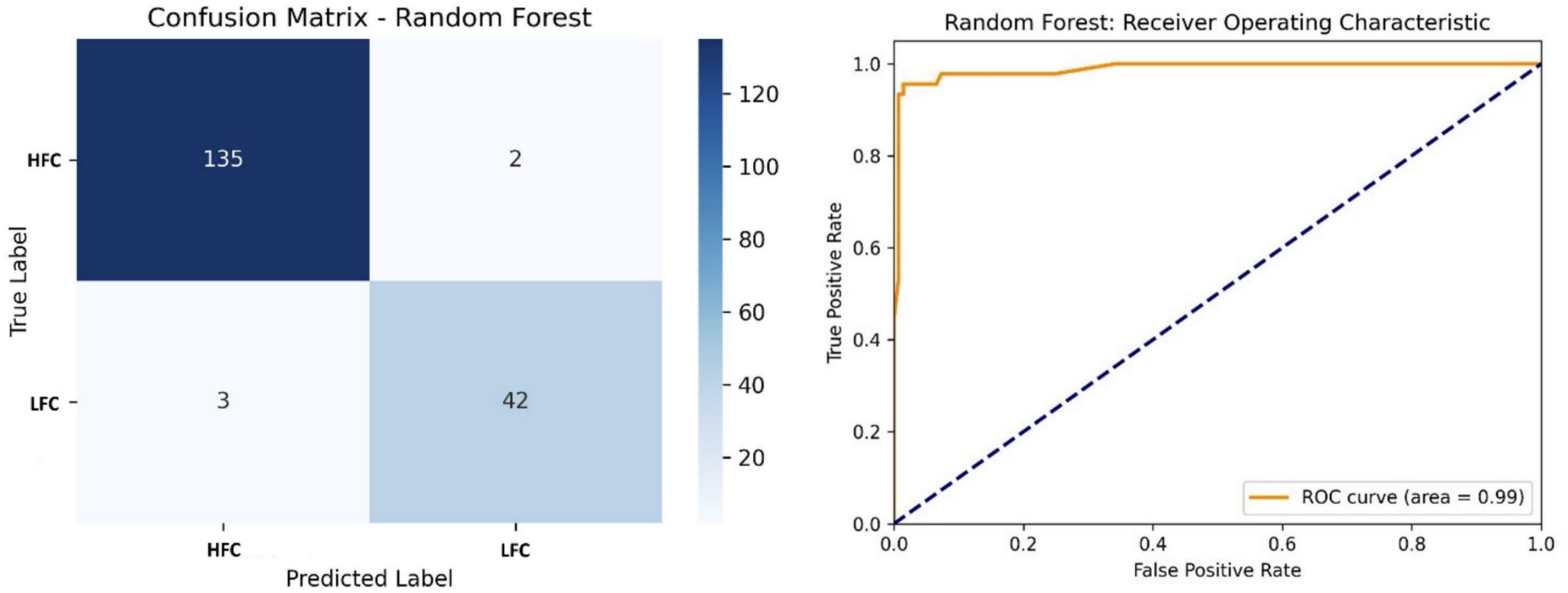
Left side – Confusion matrix of the Random Forest classifier, displaying classification accuracy of HFC and LFC calls; Right side – ROC curve of the Random Forest model, with a high AUC (0.99) confirming robust classifier performance.

The Random Forest classifier yielded strong results, correctly predicting 135 instances of distress and 42 of calm calls, with only minimal misclassifications. Achieving 97.25% accuracy and an AUC of 0.99 (Figure 8), the model demonstrated robust generalization. Its ensemble architecture allowed effective handling of feature diversity and noise.

**Figure 9:**
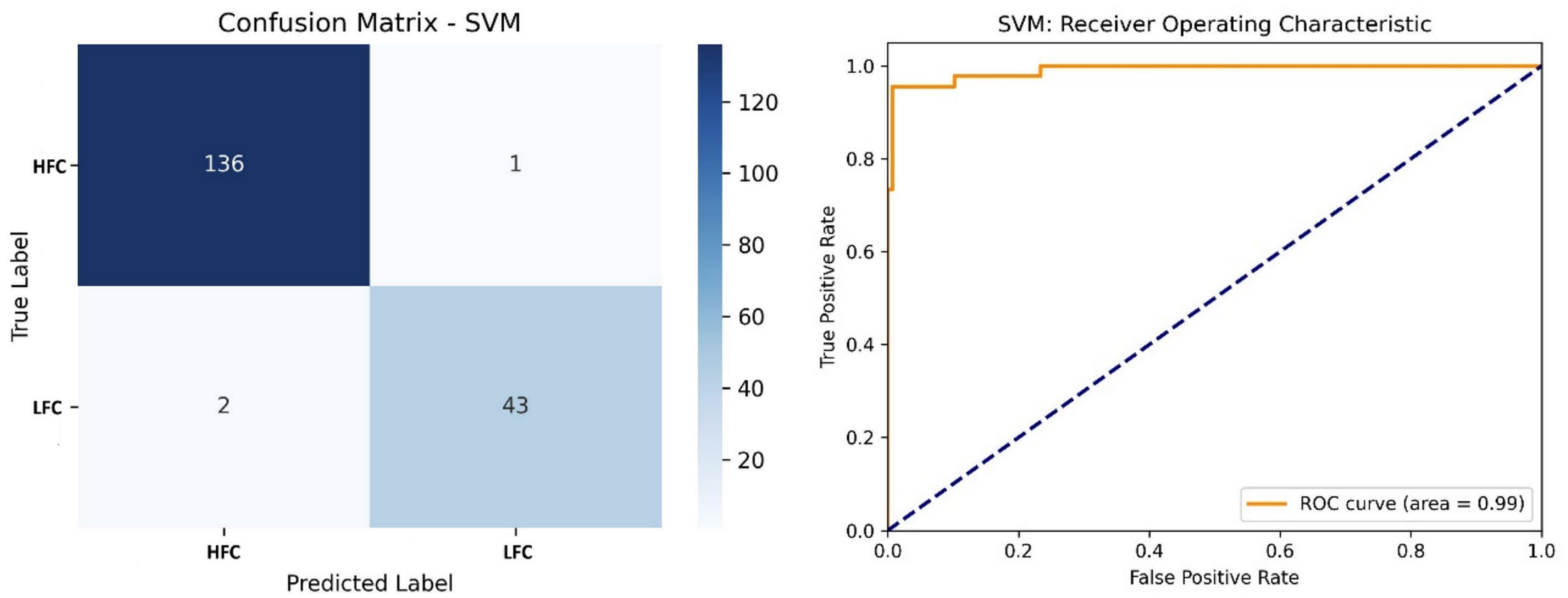
Left side – Confusion matrix of the SVM classifier, demonstrating superior accuracy in classifying call types; Right side – ROC curve of the SVM model, showing near-perfect separability of vocalization classes.

Figure 9 shows that the SVM classifier outperformed the others with an accuracy of 98.35% and an AUC of 0.99. The model correctly classified 136 HFC and 43 LFC calls, using a linear kernel to separate emotional states based on fused acoustic features.

**Figure 10:**
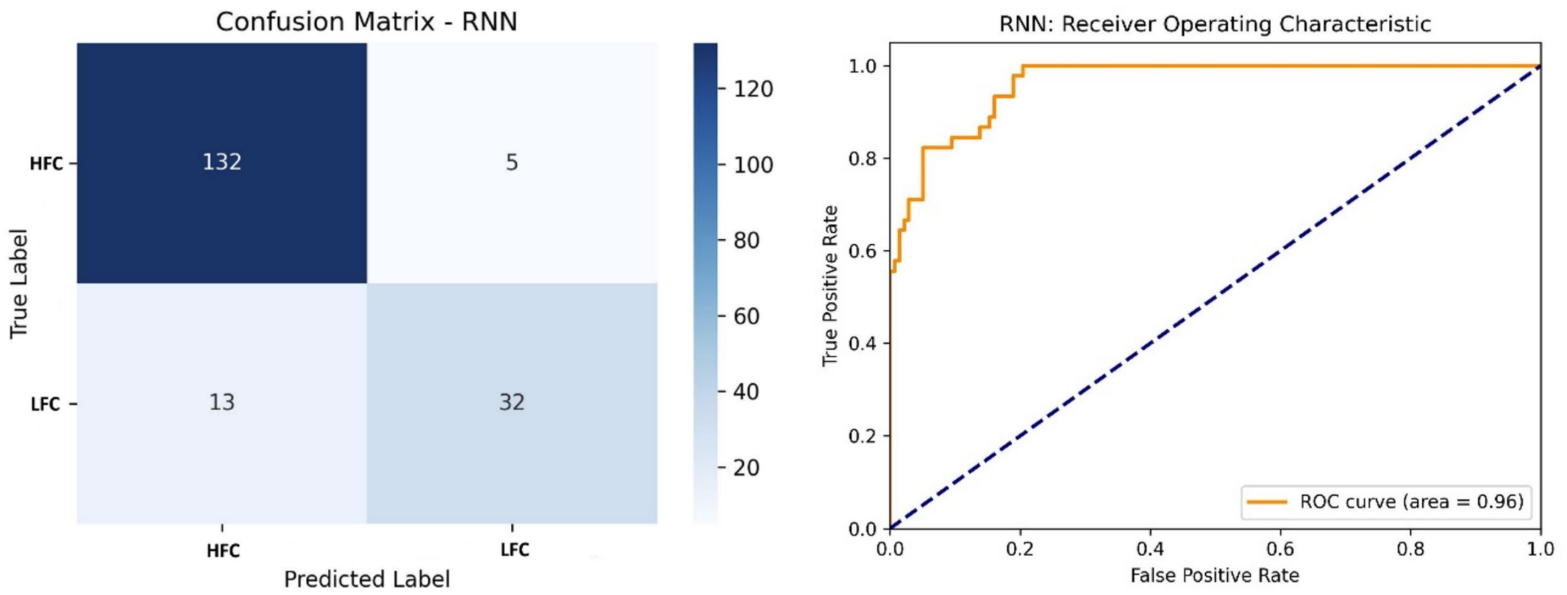
Left side – Confusion matrix of the RNN model, highlighting strengths and limitations in sequence modeling; Right side – ROC curve of the RNN model, with a moderate AUC (0.96) indicating effectiveness, with room for improvement.

The RNN model reached 88% accuracy and 0.96 AUC (Figure 10). While the model performed well on HFC classification, it struggled with LFCs, possibly due to class imbalance and sequence length sensitivity. This suggests the need for augmented training data or more complex recurrent structures.

**Figure 11:**
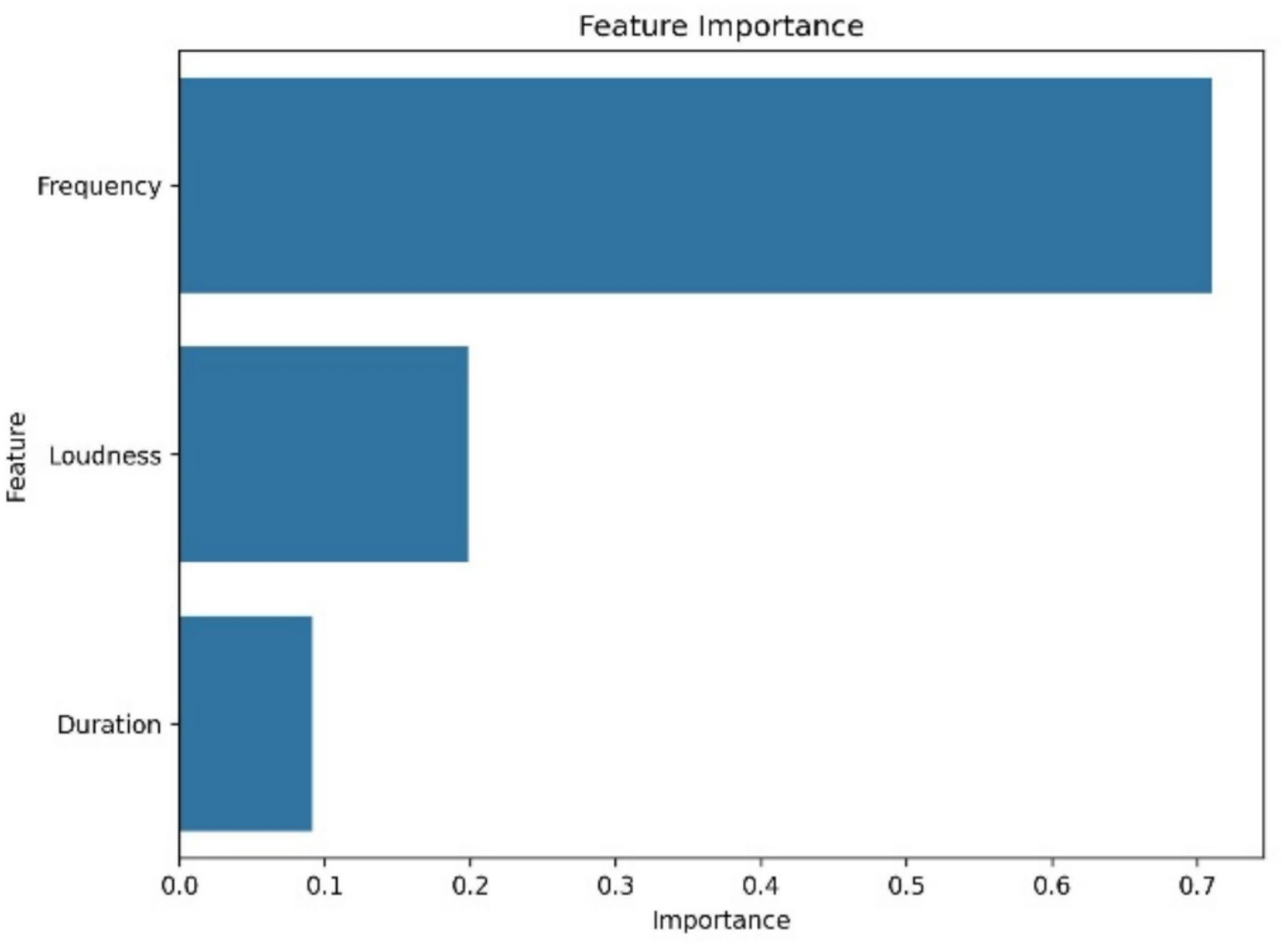
Feature importance in Random Forest model. Frequency, loudness, and duration rank as top predictors for vocalizations classification.

Table 2 provides a comparative evaluation. The SVM model achieved the highest F1-scores across both classes. Random Forest followed closely, particularly strong in detecting distress related vocalizations. The RNN lagged in LFC identification. These outcomes affirm that multi-source acoustic fusion improves model performance in cattle vocalizations classification. Figure 11 highlights the most predictive features based on Random Forest outputs. Frequency contributed most (importance score: 0.70), followed by loudness (0.22) and duration (0.09). Understanding feature impact not only aids model interpretability but also informs the design of targeted monitoring solutions (45–48).

**Table 2:** Performance evaluation result of Random Forest, support vector machine and RNN.

Model	Class	Precision	Recall	F1-Score	Accuracy
Random Forest	HFC	0.98	0.99	0.98	0.9725
	LFC	0.95	0.93	0.94	
	Macro Avg	0.97	0.96	0.96	
	Weighted Avg	0.97	0.97	0.97	
SVM	HFC	0.99	0.99	0.99	0.9835
	LFC	0.98	0.96	0.97	
	Macro Avg	0.98	0.97	0.98	
	Weighted Avg	0.98	0.98	0.98	
RNN	HFC	0.89	0.97	0.93	0.88
	LFC	0.88	0.62	0.73	
	Macro Avg	0.88	0.80	0.83	
	Weighted Avg	0.88	0.88	0.88	

The Cow Vocalization Ontology was used to conceptualize emotional state classifications. It groups vocalizations into HFCs and LFCs based on acoustic thresholds and aligns these categories with behavioral contexts observed during previous studies. The framework is built on previous acoustic-emotional mappings (2–4, 22). We acknowledge the limitation of relying solely on behavioral communication context. Future studies will integrate physiological sensors to strengthen this ontology, such as stress biomarkers and infrared thermography data. Our use of the Whisper model to extract symbolic sequences allowed for novel analysis of vocal structure. While not interpreted linguistically, the patterns, especially dominant bigrams, revealed repetitive, structured components that may signal persistent emotional states. These motifs can be integrated into future recurrent models or sequence-based behavioral classifiers, similar to methods used in studies on vocal learning species (12, 35). We emphasized that the SVM model achieved the highest performance (98.35% accuracy/F1), outperforming the other models, especially when fused acoustic and symbolic features were utilized. Additionally, we confirmed through feature importance analysis that frequency, loudness, and duration were the most predictive variables.

## 4 Discussion

In the present study, Whisper was not used to infer semantics or syntax in the human linguistic sense. Rather, it served as a pattern recognition and feature extraction tool, capable of isolating sequential acoustic motifs. Worth mentioning is that the current study does not imply that cow vocalizations possess linguistic structures such as grammar or syntax. Instead, vocal sequences were categorized as temporally organized signals that may contain biologically meaningful patterns. Hence, in this study, the notion of symbolic encoding refers not to linguistic content, but to the transformation of complex acoustic signals into symbolic representations suitable for machine learning analysis. The integration of acoustic analysis, machine learning, and symbolic sequence modeling in this study validates a powerful framework for understanding communication in dairy cows. These findings extend earlier research in animal bioacoustics and demonstrate that vocal cues can be reliably analyzed using computational approaches. The evidence presented across spectral, temporal, formant, and prosodic dimensions demonstrates that HFCs and LFCs carry distinct acoustic signatures. These vocal features, especially frequency, amplitude, and duration, were confirmed as dominant predictors of emotional state (49, 50). The high performance of both SVM and Random Forest models in classifying HFC and LFC calls demonstrates the practical feasibility of deploying such systems on-farm. Symbolic motif analysis adds a new layer of granularity, revealing structural patterns in vocalizations that correlate with stress responses. By incorporating these motifs into behavioral classifiers, future systems can achieve greater accuracy and adaptability. Moreover, the framework holds promise for broader applications in cross-species emotional modeling and neuroethology. Furthermore, the emergence of repetitive acoustic motifs, such as recurring bigrams or trigrams, aligns with theories from information theory and bioacoustics (e.g., Shannon’s redundancy for signal reliability, Wiener’s signal-to-noise optimization, and ethological concepts of degeneracy and modularity supporting communicative robustness). Repetition within vocal sequences may serve to increase the salience or redundancy of signals in noisy environments, especially during periods of distress. For example, repeated “rr” or “mm” motifs in high-frequency calls may not constitute syntactic units in the linguistic sense but can nonetheless function as consistent acoustic markers of arousal or need (12, 51, 52). Comparable studies across taxa confirm that symbolic sequence modeling is an effective approach for decoding non-linguistic acoustic structure. For instance, Bosshard et al. (12) applied symbolic sequence analysis to *Callithrix jacchus* (common marmosets), revealing bigram motifs analogous to those observed in our dairy cattle dataset. Likewise, Sainburg et al. (35) demonstrated that motif-based representations capture repertoire diversity across multiple songbird species, while research on other vocal-learning primates shows that recurrent symbolic patterns reliably accompany emotional or social contexts. Despite these strengths, we acknowledge the absence of physiological validation in the current study. To address this, we have outlined plans to integrate biomarkers such as cortisol, heart rate variability, and infrared thermography in future work. Such integration will strengthen the interpretive power of our ontology and improve the biological relevance of vocal emotion classification. The path toward intelligent animal care lies in deploying emotion-aware systems directly into the infrastructure of precision livestock farming. Embedding real-time vocal monitoring into robotic milking stations, smart barn sensor networks, and commercial animal behavior platforms has the potential to transform welfare from a periodic assessment into a continuous, responsive process. Acoustic sensors positioned in milking parlors or calving pens could flag early distress, discomfort, or illness, triggering automated alerts and informing on-farm decisions with minimal human intervention. These types of sensors have been developed to predict emotional state and social isolation (18), oestrus (53), respiratory diseases (54) and painful husbandry procedures (55). This represents a critical shift from reactive to proactive animal welfare management. Building models that perform reliably across diverse farm contexts requires a broader and more inclusive dataset. Integrating recordings from multiple cow breeds, production systems, and geographical regions would improve generalizability, enabling algorithms to adapt to variation in breed-specific vocal anatomy, environmental noise profiles, and behavioral baselines. This fusion of environmental, acoustic, and behavioral data strengthens model robustness and ensures relevance in real-world deployment. To further improve model performance and sensitivity to subtle emotional states, advanced machine learning architectures such as transformers and self-supervised learning frameworks should be explored. These approaches are well-suited for capturing long-term dependencies in vocal sequences and detecting emergent patterns from sparse or imbalanced datasets. When applied to longitudinal herd data, such models can help uncover trends in stress, social dynamics, or disease progression over time. Expanding beyond audio alone, the integration of visual and behavioral data offers a multidimensional view of animal welfare (56). By fusing indicators such as gait asymmetry, tail posture, facial tension, and ear orientation with vocal features, future systems can achieve a more nuanced and reliable assessment of emotional state. These multimodal systems could be embedded in barn-mounted camera arrays or wearable sensors, enabling real-time inference and targeted interventions. The end goal is the development of intelligent, sensor-driven platforms that integrate vocal, visual, behavioral, and physiological data into a unified, real-time decision support system. These platforms could be deployed in commercial barns, robotic milking systems, or even mobile health units, providing continuous feedback on herd welfare and individual animal status. Such systems would not only enhance welfare and productivity but also improve public trust in livestock practices by providing transparent, science-based insights into animal well-being. A key limitation of this work is the relatively small dataset (20 cows, 1,144 vocalizations), collected under a single housing system. This restricts the extent to which the findings can be generalized across breeds, management systems, or acoustic environments. We clearly acknowledge this limitation and encourage future studies across different farms, breeds, and management systems to validate and extend our results. Additionally, the lack of concurrent physiological validation (e.g., cortisol, thermography) limits the direct confirmation of inferred emotional states, although behavioral paradigms provide strong indirect evidence. Another limitation of this work is the absence of formal ablation analyses, which should be incorporated in future research to better quantify the influence of individual acoustic features and model components. From a technical perspective, machine learning (ML) has driven substantial progress in automated acoustic data processing and pattern recognition across multiple fields, from speech and ocean acoustics to animal bioacoustics (57–62). In general, ML approaches fall into three main categories: supervised, unsupervised, and reinforcement learning, with the first two being the most widely used in acoustic research (63). Feature representations, whether derived directly from raw signals, reduced using principal component analysis (PCA), or modeled probabilistically through Gaussian mixture models (GMMs), are fundamental for enabling ML systems to detect and learn structure in complex acoustic data (64–67). Importantly, ML can complement traditional physics-based acoustic models by uncovering patterns that are difficult to capture analytically, supporting hybrid strategies that integrate physical insight with data-driven inference (65, 66). Nonetheless, one of the main limitations of ML, particularly deep learning, remains its reliance on large training datasets and the limited interpretability of its internal representations (63).

## 5 Conclusion

Harnessing the capabilities of machine learning and multi-modal information fusion has opened new frontiers in decoding vocal expressions in dairy cattle. By classifying calls into high- and low-frequency categories using fused acoustic features such as pitch, loudness, and duration, the framework outlined here demonstrates a practical pathway toward real-time, non-invasive welfare monitoring. Moreover, the top-ranked features identified by the models, particularly frequency, amplitude, and duration, correspond closely with behavioral indicators of arousal and welfare. The performance of Support Vector Machine and Random Forest classifiers affirms the viability of integrating such tools into future intelligent farm management systems. Translating raw audio into structured, symbolic representations using the Whisper model added a unique layer of interpretability. These representations, while not semantically decoded, captured consistent bigram patterns that enrich our understanding of vocal cues. Their integration alongside acoustic parameters supports a deeper exploration of temporal structure in animal communication. Deploying these fusion-based systems within working agricultural environments could offer transformative potential. Real-time monitoring powered by multi-sensor integration would allow for the early identification of stress, illness, or discomfort. Such proactive interventions can not only elevate welfare standards but also improve productivity, resource efficiency, and decision-making precision on farms.

## Data Availability

The raw data on cow vocalizations presented in this study can be found in online repositories. The names of the repository/repositories and accession number(s) can be found here: https://gitlab.com/is-annazam/bovinetalk.

## Supplementary material

SUPPLEMENTARY DATA SHEET 1 Supplementary materials supporting reproducibility and implementation of the study, including: CowVocalizationImplementation.ipynb - Jupyter notebook outlining the model training and evaluation pipeline. cowvocalizationextraction.ipynb - Jupyter notebook describing the acoustic feature extraction workflow. CowVocalizationOntology.yaml - Structured ontology file mapping acoustic parameters to emotional states. combined_transcriptions.xlsx - File containing Whisper-generated vocalization transcriptions linked to acoustic features.
